# Topographic controls of soil organic carbon on soil-mantled landscapes

**DOI:** 10.1038/s41598-019-42556-5

**Published:** 2019-04-23

**Authors:** Nicholas R. Patton, Kathleen A. Lohse, Mark S. Seyfried, Sarah E. Godsey, Susan B. Parsons

**Affiliations:** 1 Department of Geosciences, Idaho State University, Pocatello, ID 83209 USA; 2 Department of Biological Sciences, Idaho State University, Pocatello, ID 83209 USA; 3 Agricultural Research Service, Northwest Watershed Research Center, 800 Park Blvd., Plaza IV, Suite 105, Boise, Idaho 83712 USA; 4 Present Address: School of Earth and Environmental Sciences, University of Queensland, St Lucia, 4072 Queensland Australia

**Keywords:** Carbon cycle, Carbon cycle

## Abstract

Large uncertainties in global carbon (C) budgets stem from soil carbon estimates and associated challenges in distributing soil organic carbon (SOC) at local to landscape scales owing to lack of information on soil thickness and controls on SOC storage. Here we show that 94% of the fine-scale variation in total profile SOC within a 1.8 km^2^ semi-arid catchment in Idaho, U.S.A. can be explained as a function of aspect and hillslope curvature when the entire vertical dimension of SOC is measured and fine-resolution (3 m) digital elevation models are utilized. Catchment SOC stocks below 0.3 m depth based on our SOC-curvature model account for >50% of the total SOC indicating substantial underestimation of stocks if sampled at shallower depths. A rapid assessment method introduced here also allows for accurate catchment-wide total SOC inventory estimation with a minimum of one soil pit and topographic data if spatial distribution of total profile SOC is not required. Comparison of multiple datasets shows generality in linear SOC-curvature and -soil thickness relationships at multiple scales. We conclude that mechanisms driving variations in carbon storage in hillslope catchment soils vary spatially at relatively small scales and can be described in a deterministic fashion given adequate topographic data.

## Introduction

Soil carbon (C) is a source of large uncertainty in both C cycling and global climate models^1–6^ with current global soil C estimates ranging from 1500–2400 Pg C^7^. Much of this uncertainty arises from the challenge of upscaling often highly spatially and temporally heterogeneous soil C stores measured at point scales to the landscape and global scale^8–10^. In particular, distributing soil organic carbon (SOC) stocks is difficult in complex terrain because of considerable heterogeneity associated with topography and soil thickness. Indeed, investigations of total catchment SOC inventories rarely quantify soil thickness despite a growing number of studies pointing to its importance in driving hydrologic and biogeochemical processes^11^ and deep (>1 m) soil carbon storage^12–14^. The lack of SOC stock data at depths >1 m mostly reflects the physical and monetary cost of excavating soil below this depth. New methods that can predict variations in SOC inventories across complex terrain would improve estimates of carbon at the local to global scales^13,15,16^.

Typical methods for estimating the spatial distribution of SOC stocks are based on soil taxonomic units^17,18^, vegetation units^13,19^, and increasingly on environmental covariates such as vegetation cover^20^ and terrain attributes such as slope, elevation^21^, hillslope position^22–24^, and hillslope shape as measured by curvature^25^. Multiple regression, random forest modeling, and other methods have been employed and combined with standard geostatistical methods such as co-kriging to predict soil properties^20,26–32^. Given the physical and monetary constraints of collecting these data, most methodologies quantify SOC stocks to specific depths, typically 0.3 m or 1 m, or extrapolate vertically from near-surface measurements^13,20,28,29,32^. Yang *et al*.^32^, for example, used remotely sensed vegetation indices, climate, and elevation covariates in combination with soil depth functions to derive a three-dimensional distribution of SOC inventories to 1 m depth for the entire Tibetan Plateau. Despite advances in relating SOC stocks to remotely sensed vegetation indices and terrain attributes, methods accounting for subsoil SOC stocks (>0.3 m) and the role of aspect and topography in controlling its distribution remain critical needs. Topographic controls may be especially important in dryland systems, where microclimate and redistribution of water, and thus soil thickness, are often strongly related to topographic characteristics^33–35^.

Dryland regions, which comprise over 40% of the terrestrial earth surface^36^, store considerable soil carbon^9^ and are considered to be some of the most responsive environments to global climate changes^37^. Recent studies show marked underestimation bias and differences among global climate models with respect to carbon (C) stocks and turnover in these regions^10,37^. Much of the uncertainty associated with these simulations may be due to under-representation of topography in global models^11^ and its influence on lateral redistribution of water and soil thickness^16^. More accurate descriptions of carbon stocks and how they interact with hydrologic processes mediating carbon turnover as well as better representation of variable soil thickness and total profile SOC inventory distributions have been identified as critical to advancing regional to global scale carbon modeling and predictions^13,37,38^.

Complex terrain, characteristic of many dryland regions, results in pronounced variation in soil thickness and SOC stocks and produces ecological and geomorphological environments that can result in microclimates rivaling broader climate controls^34,39^. Seyfried *et al*.^34^ showed, for example, that aspect-driven differences in mean annual soil temperature of 5.2 °C between north- and west-facing slopes at the same elevation were greater than the 4.4 °C difference associated with a 910-m elevation gradient spanning a 239 km^2^ semi-arid catchment. Others have documented aspect-related differences in SOC storage in drylands^20,40,41^ as well as more humid environments^42^ owing to solar insolation and vegetation differences. Hillslope features such as slope and hillslope shape (i.e., whether convergent or divergent) can also strongly control microclimate because they influence the lateral redistribution of water, soil, and nutrients through soil catena relationships^43^. In particular, hillslope curvature, which describes hillslope shape by quantifying the rate of change in slope in all directions^44,45^, has been identified as a key terrain attribute in controlling the redistribution of soil^46,47^ and consequently SOC inventories^23,48–51^. Yoo *et al*.^48,49^, for example, showed total profile SOC to be strongly associated with hillslope curvature at the plot scale (0.008 and 0.0026 km^2^) and more recently, Wang *et al*.^50^ showed similar relationships at the hillslope scale (<150 m transect). Long-term model simulations have also correlated depth-integrated SOC stocks with local curvature^51^. While this previous work relates SOC stocks to curvature at the plot and hillslope scales and in modeled domains, it remains untested whether such patterns hold at the catchment spatial scale that is useful for prediction of total SOC inventory distributions, and whether these patterns are transferable to different soil types and climates.

A recent study by Patton *et al*.^35^ introduced a simple empirical model predicting the spatial distribution of soil thickness that holds insights into extending these observations to the catchment scale. Across a diverse dataset, Patton *et al*.^35^ showed that the thickness of the mobile regolith (TMR), which describes the portion of the soil profile moved via mixing or slope processes^52^ (to which we refer simply to as soil thickness hereafter), varied linearly with hillslope curvature but the slopes and intercepts of these linear functions varied. Surface roughness–as measured by the standard deviation in catchment curvatures distributions–was associated with the slope of the linear soil thickness-curvature relations for each catchment, with increased surface roughness resulting in a decrease in the slope function towards zero. Catchment curvature distributions were also shown to be normal and centered on zero curvature (0 m^−1^) such that the most frequent (or mean) soil thickness within each catchment (i.e., the intercept for thickness-curvature relations) could be determined from a planar surface (at 0 m^−1^). These findings indicate that (1) topographic metrics of curvature are useful for predicting the entire vertical dimension of soils (not just the surface characteristics), and thus (2) topographic metrics may be more useful in the prediction of spatial distributions of SOC stocks than previously considered.

**Figure 1:**
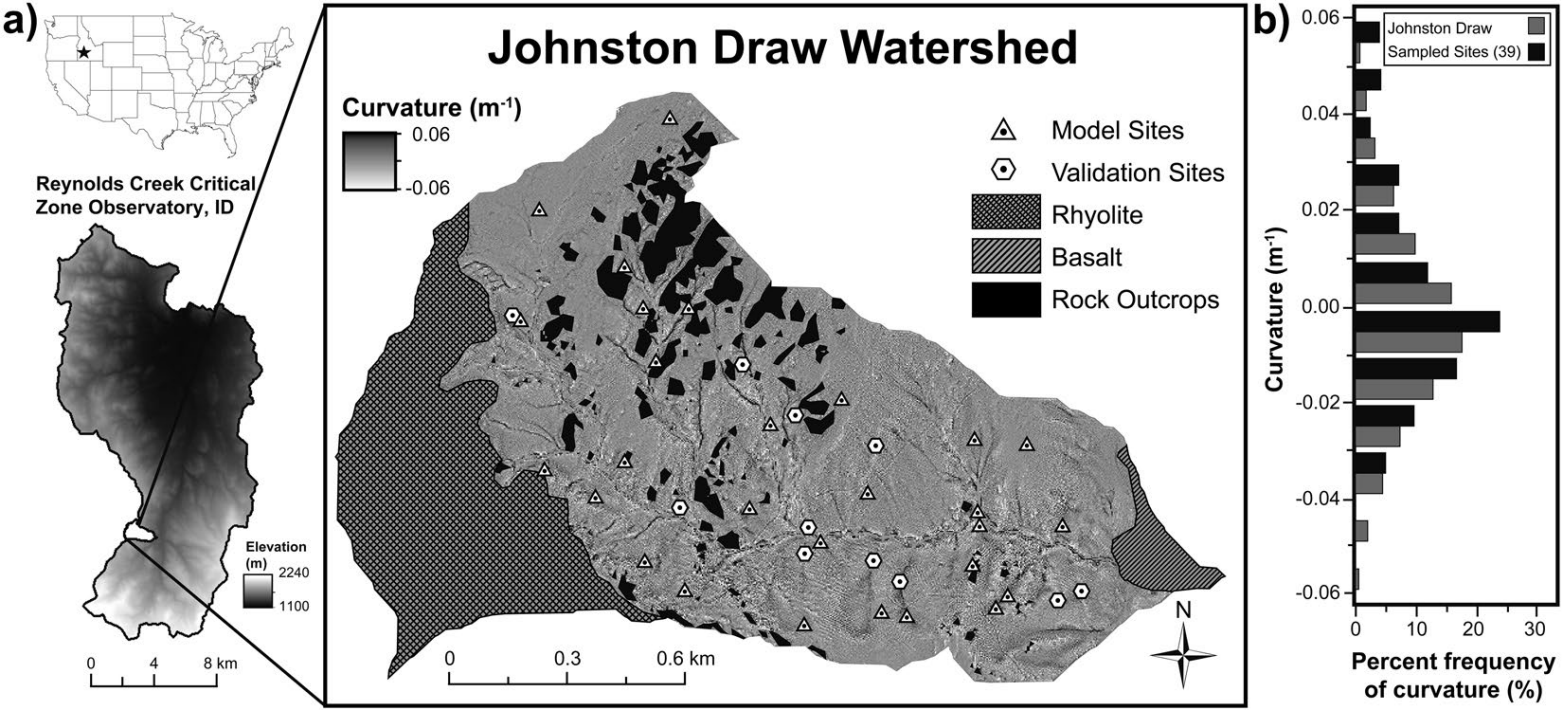
Hillslope curvature across Johnston Draw catchment. (**a**) A hillslope curvature map of Johnston Draw, a subcatchment of Reynolds Creek Critical Zone Observatory, Idaho based on a 3 m posting of a digital elevation model (DEM) derived from a 2007 Light Detection And Ranging (LiDAR) data set (10.18122/B27C77), showing the location of the model (triangles) and validation sites (hexagons) within the soil mantled, granitic portion of the Johnston Draw catchment. (**b**) Curvature histograms for sampled sites (black bins) and the entire the Johnston Draw catchment (gray bins) show normal distributions centered around 0 m^−1^, which are planar surfaces.

Here we show the generality of two methods of estimating SOC stocks at the profile to catchment scale that rely on fine-resolution topographic data and limited soil measurements. We perform similar analyses for soil total nitrogen (N) (*available in Supplementary Information*) but emphasize SOC analyses and discussion hereafter. We first describe and quantify the spatial distribution of SOC pools as it varies with respect to aspect and hillslope shape in a semiarid catchment in the Reynolds Creek Experimental Watershed (RCEW) and Critical Zone Observatory (CZO) in southwestern Idaho, USA (Fig. 1). We focused our study on Johnston Draw (JD), a 1.8 km^2^ granite dominated sub-catchment situated in the western portion of the RCEW and oriented east to west. Soil creep due to bioturbation of burrowing animals and freeze-thaw cycles are the primary soil transport mechanisms observed at JD. We hypothesized that hillslope curvature and aspect determine catchment scale distributions of SOC stocks across dryland ecosystems. Specifically, we expected that (1) north-facing aspects would contain larger total SOC stocks than south-facing aspects owing to greater moisture content and carbon inputs, and (2) convergent topography would have larger total SOC stocks than divergent topography owing to greater soil thickness and lateral transport. We tested these hypotheses by measuring total profile SOC stocks at 39 soil pits that were randomly stratified across six elevation bands and then later supplemented by 6 additional pits to represent the full range of hillslope curvatures observed in the granitic portion of the catchment. We excavated each soil pit vertically to depths below the mobile-immobile regolith (saprolite) contact and determined total profile SOC stocks. We then estimated the total catchment SOC inventories by distributing our total profile SOC stocks estimates based on two hillslope curvature-SOC functions (see *Methods and Materials*). We compared our total catchment SOC estimates to those derived from other commonly measured depths (0.05 m, 0.1 m, 0.2 m, 0.3 m, 0.5 m, and 1 m) and other common extrapolation methods. We also introduce a new rapid soil carbon estimation method, which is based on the finding of Patton *et al*.^35^ that planar surfaces represent catchment-average soil thickness, and thus SOC inventories can be determined from high-resolution topographic information and a limited number of measured soil profiles on planar surfaces, and these estimates can then be used to estimate catchment SOC stocks. Finally, we compare our findings to similar datasets^48–50,53^ from arid to mesic sites (Table 1) to evaluate the generality of topographic controls such as curvature and soil thickness on total profile SOC stocks.

**Table 1:** Site characteristics including major aspect, ecosystem, lithology, soil transport mechanism, mean elevation, mean annual temperature (MAT), and mean annual precipitation (MAP) for the cross-site analysis. ^¥^Ranges, rather than means, are provided for Reynolds Creek because of the steep gradients within this 239 km^2^ basin.

Location	Major Aspects	Ecosystem	Lithology	Soil Transport Mechanisms	Mean Elevation (m)	MAT (°C)	MAP (mm yr^−1^)	Reference
Johnston Draw, ID, USA	North and South	Semi-arid sage-steppe	Granodiorite and quartz monzonite	Animal burrowing and freeze thaw	1,600	7.4	550	This study
Reynolds Creek, ID, USA	East and West	Semi-arid sage-steppe	Granodiorite, basalt and rhyolitic welded tuff	Animal burrowing and freeze thaw	1,100–2,250	5–11	250–1,100	This study^¥^
Tennessee Valley, CA, USA	Southwest	Mediterranean coastal shrubland	Greywacke sandstone	Animal burrowing and periodic channel evacuation	170	14	1200	Yoo *et al*.^49^
Black Diamond, CA, USA	East	Mediterranean grassland	Clay-rich marine shale	Clay shrink swell	275	16	330	Yoo *et al*.^49^
Frog Hollow, NSW, AU	Southeast	Semi-arid eucalyptus savannah	Granodiorite	Animal burrowing and periodic overland flow	930	19.4	549	Wang *et al*.^50^ and Wackett *et al*.^53^
Nunnock River, NSW, AU	North	Temperate eucalyptus forest	Granodiorite	Animal burrowing and tree throw	400	22	816	Wang *et al*.^50^ and Wackett *et al*.^53^

## Results

### Variation in measured soil thickness and organic carbon content with aspect and hillslope shape

For excavated soil pits, soil thickness to saprolite on north-facing aspects varied from 0.17 m in divergent areas to 2.13 m in convergent zones, whereas thickness on the south-facing aspect varied from 0.18 m in divergent to 1.55 cm in convergent zones (Fig. 1). The average modeled thickness was not significantly different with aspects, 1.31 ± 0.56 m (n = 18) and 0.77 ± 0.34 m (n = 21) on north- and south-facing aspects, respectively (t = 0.82 < critical t_0.05,37-two tailed_ = 2.026).

**Figure 2:**
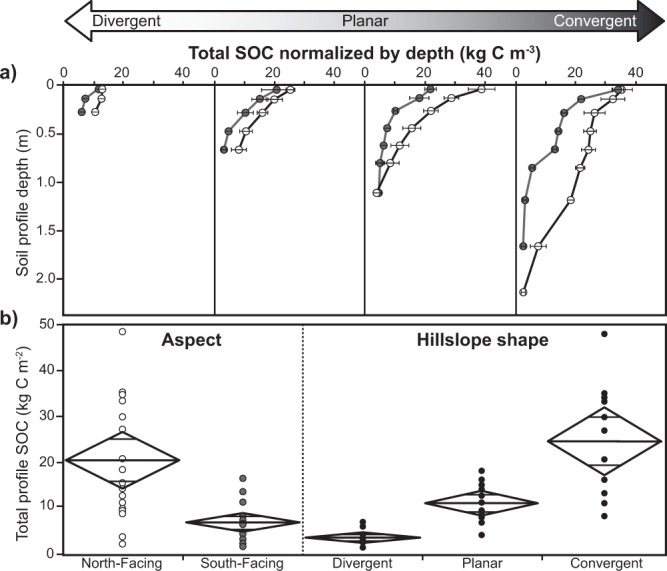
Soil organic carbon stocks vary with depth, aspect and hillslope shape. (**a**) Average total soil organic carbon (SOC) stocks normalized by depth and their associated standard errors within the Johnston Draw catchment as a function of depth and hillslope shape. Note that profiles are shallower for divergent topographic locations and deepen considerably in convergent areas for both north- (white) and south-facing (gray) aspect. (**b**) One-way ANOVA tests confirm significant differences in total SOC for aspect and hillslope topography for all excavated soil pits.

All soil profiles showed a roughly exponential decline in SOC density (kg C m^−3^), the total SOC stock normalized by the thickness interval through the entire depth profile from the surface to the mobile-immobile regolith interface (Fig. 2a). Consistent with our expectations, north-facing sites contained significantly higher total profile SOC stocks (Fig. 2b) than south-facing sites, 20.5 ± 2.24 kg C m^−2^ compared to 6.98 ± 2.12 kg C m^−2^, respectively (F ratio = 19.3, p > 0.0001). Also consistent with our expectations, convergent areas contained significantly larger total profile SOC stocks than divergent regions: 24.90 ± 2.07 kg C m^−2^ compared to 3.89 ± 1.99 kg C m^−2^, or 6.4 times more (F ratio = 27.24, p < 0.0001) (Fig. 2b).

**Figure 3:**
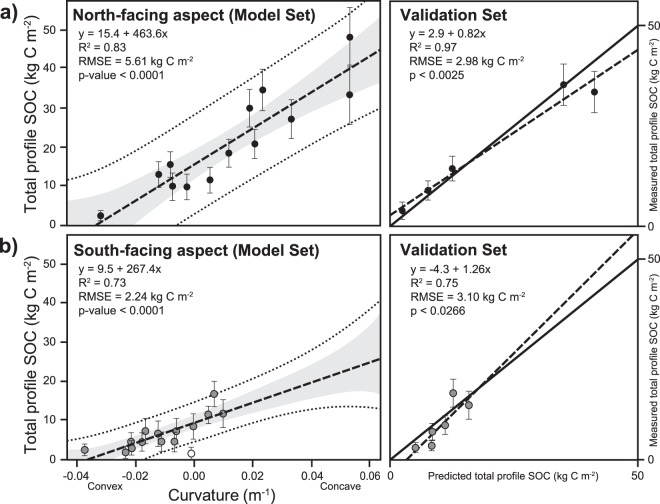
Total soil organic carbon stocks vary as a linear function of curvature on north- and south-facing aspects. Total soil organic carbon (SOC) stocks as a function of curvature for north- (black dots (**a**)) and south-facing (gray dots (**b**)) aspects, depicting a linear relationship (dashed line) bounded by 95% confidence (shaded area) and 95% prediction intervals (dashed lines). Curvature-SOC functions were derived from a model set consisting of 70% of all sites. Note one site (white dot) was excluded from the analysis owing its proximity to the stream channel and rock outcrops. The remaining 30% of sites were utilized as a validation set and evaluated against a 1:1 line (solid line) in predicted versus measured graph (dashed line).

Total profile SOC stocks varied as a positive linear function of curvature and co-varied with north- and south-facing aspects (n = 13, R^2^ = 0.83, RMSE = 5.61 kg C m^−2^, p < 0.0001 and n = 14, R^2^ = 0.73, RMSE = 2.24 kg C m^−2^, p < 0.0001, respectively) (Fig. 3). SOC validation sets showed that predicted total-profile SOC agreed well with observed values for both north- and south-facing aspects (north: n = 5, R^2^ = 0.97, RMSE = 2.98 kg C m^−2^, p < 0.0025 and south: n = 6, R^2^ = 0.75, RMSE = 3.10 kg C m^−2^, p < 0.0266, Fig. 3) with no significant difference in slope from 1 (t = 0.25 < critical t_0.05,4_ = 2.78 and t = 0.11 < critical t_0.05,5_ = 2.57, respectively). Similar agreement existed for hillslope curvature-soil total N relationships (Supplementary Fig. S1).

### Spatial distribution of soil organic carbon

**Figure 4:**
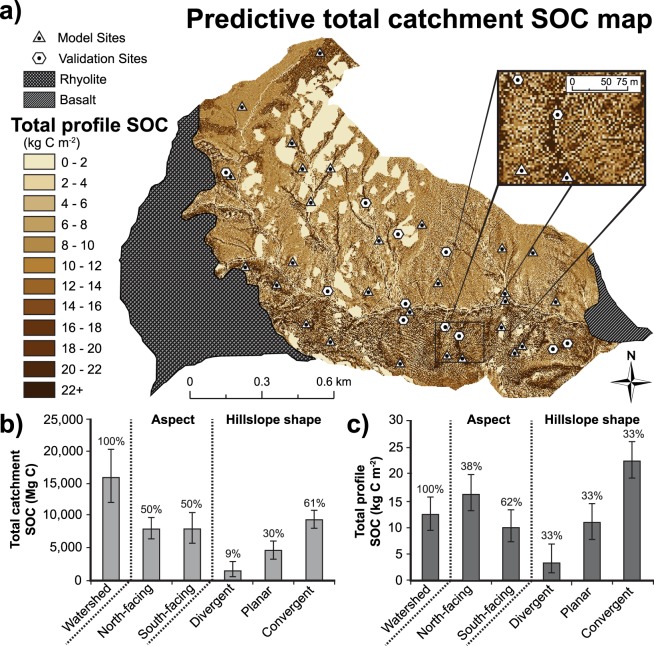
Total soil organic carbon stocks distributed across the catchment. (**a**) Estimates of total SOC stocks were extrapolated across the granitic portion of the Johnston Draw catchment using a 3 m DEM and the curvature-SOC functions developed from a model set (triangles), and were tested against a validation set (hexagons). The predictive map suggests visually that total SOC stocks vary with aspect and hillslope shape. Total SOC stocks are low on ridges and noses (light tans) and increase towards hollows and valleys (dark browns). Bar graphs represent the estimated total catchment SOC inventory (**b**) and total profile SOC stocks (**c**) for Johnston Draw with the upper 95% and lower 95% confidence limits based on relationships presented in Fig. 3. Percentages depict proportions of total SOC (**b**) and total land area (**c**) in each category (e.g., aspect or hillslope shape) for the entire catchment.

We used the two SOC-curvature functions (Fig. 3) to quantify total profile SOC stocks for each 3 m cell in the JD catchment. Figure 4 illustrates the spatially distributed catchment total SOC inventory estimate for JD based on the SOC-curvature approach; the average total profile SOC stock was 12.3 kg C m^−2^ (range: 9.4–15.7), and the total catchment SOC inventory was 15,807 Mg C (range: 12,060–20,173) (Table 2). North-facing aspects had proportionally more total catchment SOC per area, depicted by darker browns, compared to south-facing slopes, depicted by lighter tans (Fig. 4). Indeed, north-facing aspects contained 50% of the total catchment SOC despite comprising only 38% of the granitic portion of the catchment (Fig. 4b,c). Convergent topography comprised 61% of the total catchment SOC inventory (9,417 Mg C, mean 22.5 kg C m^−2^) (Fig. 4b and c) whereas divergent topography made up a smaller fraction at 3.4% (1,411 Mg C, mean 3.4 kg C m^−2^). These values compare to catchment average total profile SOC stocks on north-facing aspects that were approximately 3 times higher than south-facing aspects, and catchment average total profile SOC stores in convergent areas that were greater than 6 times than divergent hillslopes (Fig. 4b and c). Estimated catchment SOC inventories to 0.05 m, 0.1 m, 0.2 m, 0.3 m, 0.5 m, and 1 m depths represented 11.2, 20.8, 37.0, 48.4, 66.0, and 90.8% of the total catchment SOC inventory, respectively (Table 2). The large quantities of carbon found at depth indicate substantial underestimation of total catchment SOC inventories if SOC is only sampled at shallower depths (Table 2). This is also true for spatially distributed estimates of total nitrogen (N) (Supplementary Fig. 1).

**Table 2:** Summary of curvature-SOC function evaluation indices derived from commonly sampled depths and from the entire soil profile.

Interval Depth (m)	Curvature-SOC Model Validation				Catchment			
	R^2^	RMSE (kg C m^−2^)	MPE (kg C m^−2^)	SDPE (kg C m^−2^)	Average SOC (kg C m^−2^)	Total SOC Stock (Mg C)	Total SOC Stock Range (Mg C)	Relative SOC Stock (%)
0–0.05	0.28	1.22	0.51	1.45	1.4	1,777.50	2,722–903	11.2
0–0.1	0.24	1.48	0.57	1.81	2.6	3,280.90	4,866–1,801	20.8
0–0.2	0.36	1.98	1.02	1.7	4.6	5,863.50	8,379–3,466	37
0–0.3	0.38	2.97	1.19	2.4	6	7,654.10	11,133–4,459	48.4
0–0.5	0.57	3.22	1.45	2.87	8.1	10,425.40	14,599–6,644	66
0–1.0	0.88	3.27	1.59	2.85	11.2	14,355.00	18,601–10,603	90.8
Entire Profile	0.93	3.43	1.23	3.21	12.3	15,806.80	20,173–12,060	100

Indices include the coefficient of determination (R^2^), root mean squared error (RMSE), mean prediction error (MPE), and standard deviation of prediction error (SDPE). The average, total, and relative SOC inventories within the granitic portions of Johnston Draw are also reported for each depth. Relative SOC inventory is compared to the modeled total SOC inventory for the entire profile at the catchment scale. The strength of the curvature-SOC relationship increases with depth, and the typical shallow (<0.3 m) focus for assessment of SOC stocks may explain why curvature has been overlooked in previous studies.

### Model comparison to other spatial interpolation techniques

**Figure 5:**
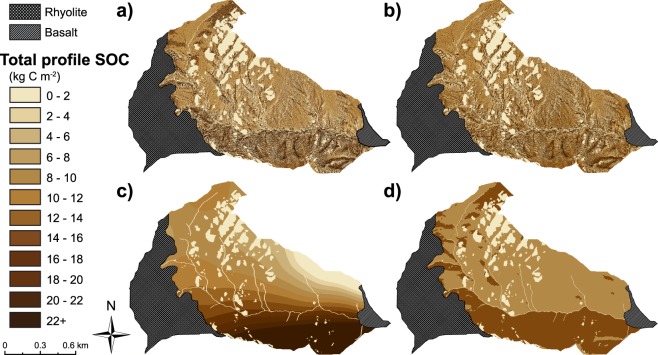
Comparison of spatially distributed total soil organic carbon (SOC) stocks using SOC-curvature method compared to kriging techniques and rapid SOC method. Spatially distributed total SOC stock estimates using the (**a**) SOC-curvature method, (**b**) regression kriging, (**c**) kriging with barriers and (**d**) the new rapid SOC method ((**a**–**d**) summarized in Table 2) within the granitic portion of the Johnston Draw catchment. SOC stocks vary from low to high values (light tans to dark browns) across the catchment. Table 3 summarizes estimated total SOC stocks, percent SOC relative to the SOC-curvature method (shown in (**a**)), and validation indices for each method.

We compared the performance of our model to other common spatial interpolation techniques such as kriging with barriers and regression kriging (Table 3) using the same available data; we purposely did not bring in additional variables with the other spatial interpolation techniques in order to test the performance of our model relative to others with similar available input data. In general, other techniques provided similar total catchment SOC estimates to our model. However, they did not distribute SOC stocks accurately, as summarized by a lower model validation R^2^ and higher RMSE and SDPE (Fig. 5; Table 3). Only regression kriging (RK), which used hillslope curvature as an environmental parameter, predicted the spatial distribution of total SOC stocks well (R^2^ = 0.82, RMSE = 4.77 kg C m^−2^). Indeed, RK model performance was much higher than other kriging methods, largely owing to its semivariogram utilizing the curvature-SOC relationship. The rapid carbon method showed good agreement with total catchment SOC inventories from the curvature-SOC approach (13,888 compared to 15,163 Mg C). However, the spatial distribution of SOC stocks based on the rapid carbon method only performed about as well as kriging with barriers. We expand on the possible practical utility of the rapid carbon method in the discussion.

**Table 3:** Summary of the total catchment SOC inventory (Mg C), percent of total SOC estimate relative to the SOC-curvature method (a), and model validation statistics in the granitic portions of Johnston Draw.

Method	Total SOC (Mg C)	Total SOC Range (Mg C)	Percent Relative to SOC-Curvature Method						Model Validation			
			Total	North	South	Divergent	Planar	Convergent	R^2^	RMSE	MPE	SDPE
SOC-curvature method (a)	15,807	20,173–12,060	—	—	—	—	—	—	0.94	3.43	1.23	3.21
Regression Kriging (b)	15,163	21,363–10,117	96	80	112	111	101	97	0.82	4.77	0.64	4.73
Kriging with Barriers (c)	17,195	28,924–6,512	109	123	95	419	108	61	0.33	9.28	2.82	8.84
Rapid SOC Budget (d)	13,888	26,677–2,774	88	93	82	336	91	50	0.24	9.73	−1.78	9.56

Spatial distributions of estimated SOC stocks are shown in Fig. 5. Higher coefficient of determination (R^2^), and lower root mean squared error (RMSE), mean prediction error (MPE), and standard deviation of prediction error (SDPE) all in units kg C m^−2^, indicate better model performance. The Rapid SOC Budget method produces realistic total soil carbon estimates for the entire catchment, suggesting that it may be a cost-effective and efficient way of estimating total SOC in a catchment, although the SOC-curvature and regression kriging methods perform better.

### Cross-site comparison

**Figure 6:**
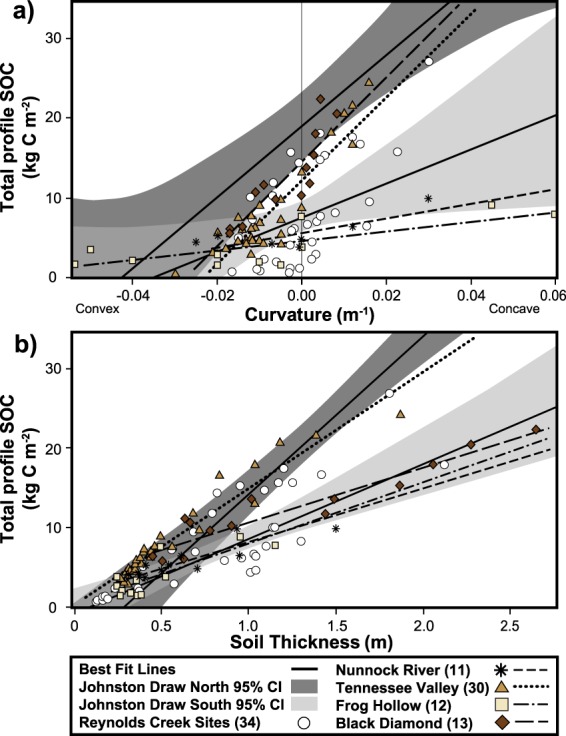
Cross-site comparison of soil organic carbon (SOC)-curvature and –thickness relations. Cross-site evaluation of four catchments and the larger Reynolds Creek Experimental Watershed (RCEW) comparing the mesic north- and xeric south-facing aspects of Johnston Draw best-fit lines (solid black lines) and 95% confidence intervals (gray shaded areas) for the relationships between SOC stocks and (**a**) curvature and (**b**) soil thickness.

Similar analyses with other comparable datasets (Table 1) revealed consistent linear relations between total profile SOC stocks and curvature (Fig. 6a) as well as SOC stocks and soil thickness (Fig. 6b); however, y-intercepts and slopes varied. We note that these comparisons were conducted using 5 m resolution digital elevation model (DEM) because most other datasets were collected at this scale. Interestingly, all SOC-curvature data fell within an envelope bounded by JD-south and JD-north (Fig. 6a). Black Diamond (BD) and Tennessee Valley (TV) SOC-curvature slope functions were not significantly different than JD’s north-facing function (t = 0.47, p = 0.64, df 27 and t = 0.46, p = 0.65, df 44, respectively) whereas Frog Hollow (FH) and Nunnock River (NR) SOC-curvature slope functions were not significantly different than JD’s south-facing slope function (t = 1.35, p = 0.19, df 27 and t = 1.71, p = 0.098, df 28, respectively). In contrast, BD and TV SOC-curvature intercepts, 14.53 ± 0.84 and 12.16 ± 0.59 kg C m^−2^ respectively, were marginally to significantly different than the JD-north intercept of 18.68 ± 2.02 kg C m^−2^ (t = 1.89, p = 0.06, df 27 and t = 3.08, p = 0.004, df 44). FH and NR intercepts, 4.39 ± 0.58 and 5.56 ± 0.53 kg C m^−2^, were significantly different than the JD-south intercept of 7.602 ± 0.92 (t = 2.95, p = 0.006, df 28 and t = 1.92, p = 0.06, df 27, respectively). Total profile SOC stocks at other Reynolds Creek CZO sites dug to the mobile regolith boundary (n = 34 soil pits) fell within an envelope bounded by the JD-south and JD-north SOC-curvature relationships.

The SOC-soil thickness functions fell within a narrow range, with slopes ranging between 6.32 to 20.2 g m^−3^. Similar to the SOC-curvature relations, TV’s SOC-thickness slope function was more similar to the JD-north than JD-south function; however, lower carbon densities at TV compared to JD-north were reflected in their significantly different slopes (t = 2.47, p = 0.017, df 44). BD, FH, NR and JD-south exhibited even lower carbon densities; slopes from these sites were not significantly different from one another (t = 1.57, p = 0.127, df 30, t = 0.76, p = 0.45, df 28, and t = 1.44, p = 0.16, df 27, respectively).

## Discussion

Jobbágy and Jackson^12^ proposed that semi-arid ecosystems contain some of the deepest stores of SOC, but these authors and many others^13,20,21,26–32^ extrapolated SOC stocks to greater depths from information obtained at shallower depths. Here we quantified the total catchment SOC inventories and showed that much of the variation in total profile SOC stocks could be explained by hillslope curvature if the entire soil profile was sampled to the mobile-immobile regolith boundary (Fig. 3). Indeed, our predicted total profile SOC stocks based on the SOC-curvature relationship agreed well with observed total profile SOC stocks (R^2^ = 0.94, RMSE = 3.43 kg m^−2^) (Table 3). Whereas other studies have also shown larger total profile SOC stocks in convergent compared to divergent areas^54,55^, our study identifies fundamental relationships between total profile SOC and hillslope curvature that allow for total catchment SOC inventory predictions with high accuracy (Figs 3 and 4). Collectively, our findings indicate that the topographic metric of curvature is an excellent predictor of total profile SOC stocks.

Consistent with our expectations and other studies^20,40–42^, we found significantly higher SOC stocks on north- compared to south-facing aspects. The aspect patterns that we observed can be partially explained by higher water storage (1.4 times greater on north than south) and lower temperatures on north-facing slopes than south-facing aspects (7.5 ± 0.47 °C compared 12.5 ± 0.12 °C, mean ± SE, n = 4 and 3 micrometeorological stations) owing to decreased solar radiation and increased canopy cover^56,57^. Interestingly, Patton *et al*.^35^ estimated similar catchment-average soil thickness values for north- and south-facing aspects, 1.00 ± 0.02 m and 0.98 ± 0.02 m (mean ± std error), respectively. Although soil thickness as defined in this study does not include immobile regolith that likely extends to greater depths and varies with aspect^58^, it contains little organic carbon (0.02–0.05%). Given the similar central tendencies in coarse fraction, texture and soil thickness values throughout the catchment, these findings indicate that both aspects have the same potential volumetric carbon storage capacity^59^. The variation in total SOC inventories associated with aspect or curvature must therefore reflect differences in carbon fluxes in and out of these different spatial domains and positive feedbacks associated with higher soil moisture, greater productivity, and more carbon inputs to soil. Findings from humid^42^ and dryland studies^20,40,41^ support these inferences showing increased surface SOC storage on the north- compared to south-facing aspects.

Combined with aspect effects, our deterministic curvature-SOC approach (Fig. 3) allows us to represent local, within-aspect SOC variations due to the dependence on curvature (Fig. 4). Because it incorporates the entire soil profile in a systematic way, it allows accurate estimates of SOC stocks across the landscape and at relatively small spatial scales (3 m). Indeed, we estimated that 51.6% of the total catchment SOC inventory was found below 0.3 m indicating substantial underestimation of carbon stocks if sampled only at shallower depths. These findings are consistent with Lozano-Carcia *et al*.^41^ who found 41% of the total SOC stock below 0.25 m in their 0.75 m soil profiles. Similar to Yoo *et al*.^48,49^ and Wang *et al*.^50^, we found convergent topography contained substantially more total SOC, >60% of the catchment SOC inventory. However, unlike their studies where SOC storage was only evaluated on a singular aspect, we showed that the largest stocks of total SOC were concentrated on the north-facing slopes, specifically in the convergent regions. The north-facing aspects contained 50% of the total catchment SOC despite representing 38% of the total land area. The relatively high total SOC contents measured at all sites (1.7 to 48.1 kg C m^−2^) on relatively steep hillslopes at Johnston Draw rival values found in productive grasslands^13^ and indicate that deep SOC storage is not limited to flat, stable surfaces. Overall, these findings indicate that substantial soil carbon stocks can be associated with complex terrain and they vary spatially at relatively small scales (3 m).

The fine-resolution total profile SOC stock maps (Fig. 4) and total N (Supplementary Fig. 1) allow for development of predictions of sources of SOC and N to streams that will likely improve understanding of the linkages between soils and streams^55,60^ and prediction of hot spots in biogeochemistry^60,61^. Sanderman *et al*.^55^ and Lohse *et al*.^60^ showed, for example, that hollows or convergent areas in zero-order catchments with high SOC stocks and TN were primary sources of DOC and nitrate (biogeochemical hotspots) to streams. On a practical side, these fine-resolution SOC and total N mapping might aid in the placement of instrumentation. For example, soil solution samplers might be placed in convergent areas with low C:N ratios calculated from SOC and N maps with the expectation that higher rates of mineralization and therefore nitrification might take place in these regions. In addition, our approach generally requires fewer samples, only those required to establish the SOC-curvature functions, whereas kriging methods generally require considerably more samples for similar accuracy (>100)^62^. More generic distribution methods, such as kriging, provide reasonably accurate estimates of overall average SOC stocks. However, these models lack SOC-topographic information, “smooth” the SOC landscape, and thus fail to capture the true variability in SOC (e.g., Fig. 5). Regression kriging (RK) with curvature as a predictor variable in our study accurately captured spatial variations in SOC stocks despite a relatively small sample size, and resulted in a distribution of SOC storage similar to the curvature-SOC approach. We expect the reason that this approach was so successful is because it incorporates both the SOC-curvature relationship and the observed spatial patterns of SOC stocks in the building of the RK model. Future improvements to the model include integrating these findings with transport, erosion, deposition models^51,63^ and linking these analyses to “top-down” remote sensing techniques for detection of surface SOC pools^64,65^. We suspect that some of the scatter in our model (Fig. 3) may also be due to differences between plant and interplant spaces that can be significant in dryland ecosystems^66^. Remote sensing methods may better capture this variation and be complementary to efforts by the digital and global soil mapping community^67^.

The fact that we determined SOC stocks through the full soil profile across an entire catchment, rather than a fixed depth or along ideal hillslope transects, may partially explain why curvature has not been identified as a useful environmental variable in previous studies^46,54^. Our work is consistent with site-specific observations by Yoo *et al*.^48,49^ and Wang *et al*.^50^ of SOC-curvature and thickness relations at the plot and transect scale, respectively. However, our cross-site findings show the generality of these relations and show possible transferability across scales (Table 1). The generality of curvature-SOC relationships may have been previously overlooked because fine-resolution curvature estimates are only now becoming more widely available through more ubiquitous Light Detection And Ranging (LiDAR) flights. A sensitivity analysis conducted by Patton *et al*.^35^ showed the highest correlation between soil thickness to saprolite and curvature at the 3 m DEM resolution; soil thickness-curvature relations estimated at lower resolutions (5–30 m DEM) resulted in deterioration of these relationships. Consistent with this analysis, our cross-site comparison revealed overall weaker correlations in SOC-curvature and SOC-soil thickness compared to our initial analyses (Fig. 3) because of the 5 m resolution curvature data used for the cross-site analysis. Despite this lower resolution, we still found relatively strong patterns at a larger catchment scale and across multiple sites (Fig. 6). Because these relationships are sensitive to scale, we expect SOC stock predictions to improve as SOC-curvature analyses are conducted at the resolution that best characterizes the land surface (e.g., 3 m^2^ in the case of JD).

Our findings of similar linear SOC-curvature as well as SOC-thickness relationships across our cross-site dataset (Fig. 6) indicate that these linear relationships are more general than appreciated, though determining the mechanisms underlying these patterns remain a challenge. Yoo *et al*.^48,49^ and Wang *et al*.^50^ explored in detail the processes underlying the strong association of SOC stocks with curvature and thickness and concluded that site-specific SOC-curvature associations were related to differences in soil thickness driven by soil production and erosion. At their sites where productivity was evenly distributed across the hillslope, patterns of SOC accumulation reflected these high rates of primary productivity (inputs) and mineralization/decomposition (outputs) relative to topographic position (sedimentation in concave areas and erosion in convex areas). Yoo *et al*.^49^ concluded that erosional deposition of SOC contributed little to SOC in concave areas. While productivity and SOC accumulation are not evenly distributed across JD (Fig. 4), studies in the RCEW have measured higher rates of net ecosystem productivity in aspen than sagebrush^68,69^ (>150 g m^−2^ yr^−1^) and relatively low particulate organic carbon export (mean ~1 g m^−2^ yr^−1^ in JD) that support the idea of high biological cycling rates relative to erosion rates^48,49^. The SOC-curvature slope analyses (Fig. 6a) indicate that BD, TV and JD-north have a similar ability to increase or decrease SOC stocks per unit curvature and indicate potential steady-state of both soil thickness and SOC stocks in all of these systems. Our study design and scope of work prevent us from further evaluating the underlying mechanisms driving these patterns. Future research is merited on this topic, and further work is also needed to assess whether the JD-north line holds in wetter environments (>1500 mm mean annual precipitation) or possibly defines the maximum rate of SOC gain per curvature.

While we observed general linear relations between SOC stocks and curvature (Fig. 6a), the slope function and intercept values of these relationships varied substantially. Based on our previous findings^35^, differences in SOC-curvature slope functions may be explained by catchment surface roughness, which has been shown to be closely tied to soil thickness. We also interpret differences in the SOC-curvature y-intercept values as differences in average catchment SOC inventory, which again is closely associated to the catchment curvature distribution and soil thickness.

To further evaluate the role of thickness in driving the SOC-curvature relationship, we plotted the SOC-soil thickness relationship (Fig. 6b). The SOC-soil thickness relationship (Fig. 6b) is quite useful for normalizing SOC relative to soil thickness and thus comparing carbon density or the carbon storage capacity of systems. Whereas BD and TV have similar SOC-curvature slope functions, the BD SOC-soil thickness slope function is much lower than TV and JD-north, indicating lower carbon densities likely owing to climate differences (Table 1). In contrast, TV is similar to the JD-north relationship even though it receives twice the amount of precipitation indicating possible lithological constraints on SOC storage at this site (e.g., coarse fraction content and soil texture)^48,49^.

We posit that deviations from the SOC-curvature line may be useful indicators of whether a system is out of steady state. In contrast to our study sites that appear to have slope-dependent sediment flux, which may ultimately explain why soil thickness is curvature dependent^70^, landscapes that are undergoing episodic mass soil movements or accelerated erosion due to tillage, stream incision, gullying and landslides may experience substantial loss in SOC stocks and therefore not be in steady state^71^. In these landscapes, SOC storage may only partially be related to curvature.

It has been suggested that SOC stocks in erosive landscape positions may become replenished post-perturbation through dynamic replacement while increasing SOC stocks in depositional areas^72–74^. However, quantifying these potential C source and sink dynamics remains a challenge and is highly debated^75,76^. As such, deviations in observed SOC from the predicted curvature-SOC line may help in quantifying potential C source and sink terms in complex terrain by revealing the SOC stocks that cannot be explained by soil thickness controls alone. For example, the modeled curvature-integrated SOC curves showing non-linear increases in SOC in concave areas in Rosenbloom *et al*.^51^ may reflect additional storage of eroded SOC in these depositional areas, which is consistent with dynamic replacement.

The success of the curvature-SOC model depends on the availability of samples collected to the mobile-immobile boundary and fine-resolution (e.g., LiDAR) digital elevation data because much of the observed variability is correlated with topographic variations at approximately a 3 m scale. Currently, limited measurements of carbon to the depth of the mobile regolith (TMR) constrain the application of this model. Studies that dig to refusal, bedrock or at a predetermined depth may include or exclude portions of the mobile regolith resulting in inconsistencies in total-profile SOC estimates. In addition, soil-sampling methods such as augering may underestimate thickness in rocky soils and/or overestimate depth if penetrating fractures in bedrock. Without properly identifying the mobile regolith boundary with soil pits, the likelihood of identifying accurate topographic relationships is low.

The accuracy of our estimates also depends on the SOC-curvature functions shown in Fig. 3. These functions in other environments will likely depend strongly on catchment curvature distributions. Patton *et al*.^35^ showed that spatially distributed soil thickness can be determined from knowledge of the frequency distribution of curvature within a catchment, with narrower distributions yielding better model performances than broader ones. Similarly, uncertainty in the SOC-curvature models will likely increase as the spread in the curvature distribution increases such that the predictive capability of the model will decline in these regions. However, catchment curvature distributions can be rapidly assessed in a geographical information system and used to determine model suitability for predicting SOC from curvature. Similar to the TMR-curvature method described by Patton *et al*.^35^, the SOC-curvature method is limited to soil-mantled landscapes dominated by diffusion-like processes; rock outcrops and stream channels may result in local disturbances to controls on soil thickness and SOC, and features that will not be captured in the SOC-curvature model should be removed before landscape SOC analysis (see *Methods and Materials* for more details). Other possible limitations to the model include uncertainty in estimating SOC stocks in environments such as colluvium where it is difficult to define the basal boundary, in floodplains where materials are introduced and exhumed with disturbance events, in aeolian landscapes where atmospheric inputs drive soil development, and in glaciated landscapes where erosion or deposition may smooth the landscape. Our model has also not been tested in more stable landscapes with deeper soils, which will likely exhibit thicker immobile regolith, saprolite, which is another feature not captured by our SOC-curvature model. Tests in these environments are warranted.

Unlike the curvature-SOC methods, the rapid assessment carbon method that we report provides a new method for total catchment SOC estimates. In contrast to the curvature-SOC method that requires prior knowledge of the curvature-SOC relationship and where uncertainty in the model will likely increase as the spread in the catchment curvature distribution increases, the rapid carbon method does not rely on curvature to be a good predictor of SOC stores. Rather, this method takes advantage of observation that the catchment curvatures are normally distributed and centered around planar surfaces (0 m^−1^) such that soil thickness and thus SOC on a planar surface represents the mean thickness^35^ and mean total profile SOC per area of the catchment, respectively. Indeed, in Patton *et al*.^35^, eight diverse catchments were shown to have catchment curvatures normally distributed and centered around planar surfaces (0 m^−1^) when the catchment boundary was delineated. Application of the rapid carbon method using BD and TV intercept values (0 m^−1^) in Fig. 6a yielded 37.2 ± 2.2 tC (mean ± SE) and 97.6 ± 0.5 tC, likely within error of BD and TV estimates of 37 and 110 tC from Yoo *et al*.^49^. These findings provide further validation of the rapid carbon method. Another benefit of this method is that it requires little sampling. If no aspect differences in SOC exist, which is likely in humid regions, *one* sample can be collected through the entire vertical dimension of the soil profile on a planar surface, and this will represent the average total-profile SOC stock which can be used to estimate the catchment SOC inventory. If aspect differences are apparent, one total-profile SOC stock estimate from a planar surface for each aspect (a total of two-four soil pits) can be used to determine an unbiased estimate of the total catchment SOC inventory. Finally, although the rapid assessment cannot distribute carbon in spatially explicit way, errors in total SOC inventory estimates associated with this method are substantially smaller than kriging-based estimates (Fig. 5 and Table 3). Future application of this method may include strategic repeated sampling on a planar surface to determine changes in catchment SOC inventories in response to perturbation, remediation or environmental change. To this end, we conclude that the rapid method can provide rapid and cost-efficient total SOC stocks across complex terrain and possibly provide strategic sampling points for monitoring catchment SOC inventories responses to perturbations or remediation.

## Conclusion

Our results indicate that the mechanisms driving variations in carbon storage in hillslope catchment soils vary spatially at relatively small scales and that they can be described in a deterministic fashion given adequate topographic data. We documented two important local topographic controls on SOC stock distribution in semiarid systems: aspect, with north-facing slopes containing roughly three times the SOC concentration as analogous south-facing slopes; and hillslope curvature, which strongly controls the redistribution of water and thickness of mobile regolith and therefore SOC content. Similarly, other soil properties such as total N also vary predictively with hillslope curvature, suggesting that curvature has a potential to be a multifaceted proxy for many soil characteristics at the fine scale. Our findings also highlight that greater than 50% the total catchment SOC inventory is found at depths greater than 0.3 m, indicating underestimations in local to global SOC stocks.

The general linear SOC-curvature and SOC-thickness relationships across sites indicate that topographic properties such as curvature contain more information on soil characteristics at multiple scales than previously appreciated. That is, SOC-curvature relationships can provide a better way to distribute SOC stocks systematically across a catchment. In combination with SOC-thickness relationships, these relationships provide useful insights into soil carbon storage capacity of systems and mechanisms driving these relationships. The quick carbon assessment method introduced here and validated with other comparable datasets allows rapid and efficient estimation of catchment SOC stocks with as few as one soil pit from a planar surface to estimate the catchment average total profile SOC inventory.

We conclude that accurate spatial distributions of total carbon and nutrient stocks from the soil surface to the saprolite can be achieved with significantly less labor and cost using high-resolution elevation data and a limited set of soil thickness measurements. Our findings provide new tools and insight to investigate mechanisms driving local to regional carbon fluxes and stocks.

## Methods and Materials

### Study Area

We conducted the study at the United State Department of Agriculture, Agricultural Research Service (USDA-ARS) Reynolds Creek Experimental Watershed (239 km^2^), and Reynolds Creek Critical Zone Observatory (RC CZO), located within the Owyhee Mountain Range in southwestern Idaho, USA. We focused our study on Johnston Draw, a 1.8 km^2^ sub-catchment situated in the western portion of RC CZO and oriented east to west (Fig. 1). The mean annual precipitation is 550 mm and mean annual temperature is 7.4 °C near the bottom of the catchment. Johnston Draw falls within the rain-snow transition at elevations ranging from 1490 m to 1850 m. Precipitation typically occurs in late fall and continues throughout the winter months with relatively dry summers. Lower elevations receive precipitation as rain while higher elevations receive precipitation as snow, which accumulates in the upper basin as drifts^56^. Wyoming Big Sagebrush (*Artemisia tridentate* ssp. *wyomingensis*) and bitterbrush (*Purshia stansburyana*) are the dominant plant species representing 50–75% of the catchment, with mountain mahogany (*Cercocarpus ledifolius*), aspen (*Populus tremuloides*), and western juniper (*Juniperus occidentalis*) being minor components. The lithology is primarily ~66–62 Mya biotite muscovite granitediorite and quartz monzonite, from the Idaho Batholith^77^. The relatively spatially continuous lithology contributes to a consistent sandy loam soil texture throughout most of the catchment across curvature and depth (Supplemental Table S1) (average: 67% sand, 18% silt, and 15% clay, n = 181). Compared to texture, coarse fraction (CF) increased with depth (Supplementary Table 1) with an average CF size of 2–8 mm and percent content by volume of 15.5 (range 1.08–48.4). Minor lithologic discontinuities include a combination of quartz latite and rhyolite flows covering the high plateau at the top of the catchment, and a small olivine-rich basalt flow that extends into the outflow^78^. To control for lithology, these discontinuities were excluded from this analysis (Fig. 1).

Johnston Draw has asymmetric aspects with 38% of the landscape with north-facing aspects and the remaining 62% with south-facing aspects. The relatively steep north-facing slopes average 16.8° compared to the south-facing slopes, which average 13.9°. The south-facing slopes have larger and more prevalent rock outcrops compared to the north-facing slopes, 11.3% of the south- and 6.9% of the north-facing aspects. The dominant soil transport mechanism is slow, grain to grain soil creep through freeze thaw and bioturbation by burrowing animals which is supported by a lack of landslides. Soils on the north-facing aspects are generally classified as Haploxerolls and Xeropsamments and Xerorthents on the south-facing aspects. Typical soil profiles characteristic of north- and south-facing aspects are shown in Supplemental Table S1. In brief, the north-facing aspect profiles have a prominent series of dark A horizons, diagnostic of Mollisols, averaging 1 m but thickening in convergent zones to 1.75 m. Bioturbation by burrowing gophers and other animals is present through the A horizons; large macropores (~9 cm in diameter) filled with high organic matter content can often be observed on north-facing aspects. On south-facing aspects, less bioturbation is typically present, and soil profiles show less vertical variation in physical properties. There is little evidence of legacy landslides in the catchment; soil movement is primarily dominated by soil creep.

### Study design and sampling

We initially randomly stratified site selection across six elevation band strata within the catchment and then later added sites to represent the full range of hillslope curvatures. We used a 3 m posting of a digital elevation model (DEM) derived from a 2007 Light Detection And Ranging (LiDAR) data set (10.18122/B27C77) based on a sensitivity analysis described in Patton *et al*.^23^. We determined the location of soil pits using Real-Time (RTK) satellite-based Global Positioning Systems (GPS) (Topcon HiPer II, Tokyo, Japan) with an accuracy of less than 1 m. We determined thickness of the mobile regolith based on protocols described by others^54,55^. In brief, we vertically excavated thirty-nine 1 m by 2 m soil pits to depths below the mobile-immobile regolith contact; we designated the lower boundary, the mobile-immobile regolith contact, based on the observation of original parent material structure including exfoliation sheets, planar flow fabrics, and jointing sets^56^.

We performed basic soil descriptions on the soil pits using standard survey methods^57^. We then collected bulk soil pedon samples (~4 L) for laboratory analysis and basic soil characteristics based on the rocky nature of native dryland soils^79^. Initially, we sampled 30 representative soil pits and collected them based on genetic horizon. Due to the lack of horizonation and large variability in percent carbon measurements, we collected the remaining 9 sites by predetermined depths intervals: 0–5 cm, 5–10 cm, 10–20 cm, 20–30 cm, 30–50 cm, and every 25 cm increment thereafter. We tested for differences in method collection used (horizon versus depth interval) on the surface horizon (0–0.3 m) where we would have expected the greatest difference between methods, and the null hypothesis of equity could not be rejected for both north- and south-facing aspects.

### Percent coarse fraction by volume

We dried bulk soil pedon samples and sieved them through a 2 mm stainless steel sieve. We separated the fine fraction (<2 mm) from the coarse fraction (>2 mm) to determine percent coarse fraction by weight. If variations in CF content are not accounted for, large uncertainties in SOC stocks may occur; however, uncertainties in percent CF and FF were low, ~5%, due to the large volume of bulk soil samples collected. We then converted coarse fraction by weight to volume using the mean coarse fraction density of 2.3 g cm^−3^ (S.D. ± 0.14), based on water displacement of coarse fraction material on >150 samples^80^.

### Percent SOC and total nitrogen (TN)

We prepared a sub-sample (20 g) of the fine fraction for percent SOC and TN analysis following standard protocols^81^. In brief, we removed roots, ground sample in a stainless steel ball grinder, packed and weighed 15–60 mg in tin capsules on a 6 pt microbalance, and analyzed them on a Costech ECS 4010 elemental analyzer interfaced to a Thermo Delta V Advantage continuous flow isotope ratio mass spectrometer (EA IRMS)(≤±0.2 standard deviation) at the Idaho State University’s Center for Archaeology, Materials and Applied Spectroscopy (CAMAS). Standards of ISU Peptone, Costech Acetanilide, and DORM-3 are calibrated against international standards (IAEA-N-1, IAEA-N-2, USGS-25, USGS-40, USGS-24, IAEA-600), and were used to create a two-point calibration. We tested all samples for soil inorganic carbon (SIC) by (1) applying several drops of 10% hydrochloric acid to determine effervescence, (2) evaluating δ^13^C values, and (3) analyzing a subset of samples for SIC using a modified pressure-calcimeter method^60^. We found no evidence of soil inorganic carbon in any samples in this study.

### Fine fraction bulk density

We randomly collected fine fraction bulk density (BD_FF_) core samples within all soil pits utilizing the short core extraction method^82^. We hammered sample cores, approximately 5 cm in diameter by 5.5 cm in height, vertically down and extracted them using a soil knife. In all, 95 bulk density measurements were collected at depths ranging from 0 to 1.22 m representing the entire soil profile from the surface to the mobile-immobile regolith contact. We oven dried all bulk density cores for 24 hours at 105 °C and sieved them through a 2 mm stainless steel sieve in the laboratory to remove and determine the fine fraction (<2 mm) and coarse fraction (>2 mm) by mass (M_FF_ and M_CF_, respectively). The combined weight of both the M_FF_ and M_CF_ is the total mass (M_T_) in the core volume (V_T_). The BD_FF_ was calculated using Eq. 1, where the volume of the V_CF_ was determined from water displacement and the V_CF_ was subtracted from V_T_ to calculate V_FF_.

1 $$B{D}_{FF}=[\frac{{M}_{FF}}{{V}_{FF}}]=[\frac{{M}_{T}-{M}_{CF}}{{V}_{T}-{V}_{CF}}]$$

To accommodate for 86 missing *in situ* measurements of BD_FF_ (out of 181 total soil samples), we adopted the protocol by Patton *et al*.^80^. In short, the felsic equation (Eq. 2) utilizes percent SOC content for a given sample interval to total bulk density (BD_T_). BD_T_ estimates were then adjusted by removing site-specific CF mass and volume in the soil pedon sample to provide

2 $$B{D}_{T}\,=\,1.413\,\pm \,0.009\,\times \,\,{SOC{\rm{ \% }}}^{-0.156\pm 0.008}$$

robust BD_FF_ estimates with a mean error of 0.092 g cm^−3^.

### Determining total profile SOC

We determined total profile SOC (g/cm^2^) for each profile from Eq. 3:

3 $${Total}\,{Profile}\,{SOC}=\sum _{i=1}^{k}\,B{D}_{F{F}_{i}}{C}_{i}{D}_{i}(1-{S}_{i})$$

where there are k soil layers, C_i_ is the organic carbon concentration (g SOC g dry soil^−1^), D_i_ is the thickness of horizon (m), and S_i_ is the coarse fraction by volume (m^3^ m^−3^)^17^. The underlying weathered granite parent material contained 0.05% carbon, and we subtracted this value from all samples to correct for background signal of bedrock-sourced carbon. Uncertainties in total profile SOC were determined by propagating errors determined for C_i_ (0.2 g SOC g dry soil^−1^), D_i_ (0.05 m), and S_i_ (0.092 g cm^−3^). We determined the average total SOC for depth intervals, 0–0.1 m, 0.1–0.2 m, 0.2–0.4 m, 0.4–0.6 m, 0.6–0.8 m, 0.8–1.0 m, 1.0–1.5 m, and >1.5 m and evaluated patterns in TOC with depth by aspect and topographic region. For aspect, we classified north-facing sites as those between 270° to 90° and south-facing sites as those between 90° to 270°, and we classified topography into divergent, planar, and convergent topographic regions based on a local hillslope curvature and aspect values that were derived from a geographical information system (GIS) (ArcGIS Map, Riverside, CA). Curvature was calculated as the rate of change in slope (as a percentage) from a fixed point relative to eight neighboring cells^44,45^ using geographical information system (ArcGIS v.10.2.2, ESRI, Redlands, CA). Note, the primary output of ArcGIS curvature values are derived from Moore *et al*.^45^ and Zevenbergen *et al*.^83^, which use the negative curvature and percent convention. Like Patton *et al*.^35^, here we adjust ArcGIS primary output by −100 to retain consistency with the geomorphic literature. We divided the curvature distribution into divergent (>−0.01 m^−1^), planar (0.0085 to −0.01 m^−1^), and convergent (<0.0085 m^−1^) regions. Each of these regions represents approximately one-third of the total land area. We performed one-way ANOVA tests to determine statistical significance of aspect and topographic regions using JMP Pro 12.1.0 statistical software (JMP, Marlow, Buckinghamshire, UK).

### Curvature- SOC relationships

We separated all sites into north- or south-facing sites as described above and then randomly selected 70% of the north- and south-facing site locations to evaluate the relationship between the hillslope curvature and total SOC for each aspect. We used a linear regression function with curvature as the explanatory variable and total SOC as the response variable. Uncertainty in curvature was propagated vertical based on reported LiDAR flight metadata according to the equations from Moore *et al*.^45^ and Zevenbergen *et al*.^78^. Standard error was determined using the Method of Moments by varying the correlation in uncertainty between elevations of adjacent cells between −1 and +1. In this analysis, we assumed correlation between uncertainty of neighbor and center cell points was 0 (r = 0). Hillslope curvature uncertainty was calculated for a 3 m and 5 m DEM, 0.0169 m^−1^ and 0.0061 m^−1^, respectively. One soil pit was considered an outlier due to its close proximity to an incised channel and rock outcrop. We used the remaining 30% of pit locations as a validation set to evaluate the goodness of fit of the model. Predicted total SOC and their confidence intervals (95%) were compared to the observed total SOC. To evaluate the spatial distribution of total SOC within the catchment, we applied the two linear functions for each aspect to a 3 m DEM curvature using ArcGIS Spatial Analyst “raster calculator” tool. We determined the total sum of the carbon stocks for the entire catchment, north- and south-facing aspects, and divergent, convergent and planar topography using ArcGIS Spatial Analyst “zonal statistics” tool. We limited our research to the hillslope portions of the catchment dominated by diffusion-like processes such that all rock outcrops and stream channels (accumulation area ≥6000 m^2^) were not included in the estimates of total SOC inventories.

### Determining catchment total SOC at commonly sampled depths

To determine total SOC stocks found at predetermined depths across the entire Johnston Draw catchment, we calculated SOC stocks for all model and validation soil pits at 0.05 m, 0.1 m, 0.3 m, 0.5 m, and 1.0 m. We then built curvature-SOC relationships for north- and south-facing aspects from model sites for each depth. We assumed that the curvature-SOC functions built from 0.05 m, 0.01 m, 0.3 m, 0.5 m, and 1.0 m represented only the total SOC stocks found at those specified depths. We evaluated each function with the validation set using four evaluation indices: coefficient of determination (R^2^), root mean squared error (RMSE), mean prediction error (MPE), and standard deviation of prediction error (SDPE). MPE is used to describe the magnitude of over- (positive values) or under-estimation (negative values). SDPE represents the variability in the predictions after correcting for MPE. RMSE describes the overall error in the predictions. Validation sets with the lowest absolute RMSE, SDPE, and MPE values are ideal, and R^2^ closer to 1 are preferred.

### Comparing methodologies for distributing total SOC

We compared our bottom-up SOC-curvature approach against other commonly used extrapolation methods, kriging with barriers and regression kriging, as well as the rapid total SOC budget approach presented here. We assessed these techniques using data through the entire soil profile. We compared the total estimates of SOC inventories for the entire catchment, north vs. south-facing aspects, and local curvature, and evaluated the spatial distribution across Johnston Draw using the same evaluation indices described in the previous section. We used the same 28 soil pits to build the kriging models and then evaluated all predictive models using the same independent validation subset (11 soil pits). Kriging with barriers, performed with ESRI ArcGIS Spatial & Geostatistical Analyst (https://www.esri.com/en-us/home), used a non-Euclidean interpolation approach that specified rock outcrops as barriers between measured locations and predictions, thus excluding them from the analysis, and a Gaussian kernel function based on lowest root mean squared errors (RMSE) as a smoothing factor. The regression kriging model’s semivariogram was generated in R studio (Boston, MA) using the previously established relationship between hillslope curvature and total SOC, and subsequently universal kriging was used to interpolate soil carbon predictions informed by this relationship.

Lastly, the rapid SOC budget approach takes advantage of observation that the curvatures are normally distributed and centered around planar surfaces (0 m^−1^). Thus, for each aspect, a single total profile SOC estimate from a planar surface represents the average total profile SOC stock for an area of interest. We selected two pits of identical TMR (1.04 m) located on planar surfaces of both the north- and south-facing aspects. The north-facing site had 15.47 kg C m^−2^ while the south-facing site had 8.35 kg C m^−2^. These values were distributed evenly across their respective aspects. The rapid carbon budget approach provides a quick and conservative estimation of the SOC stock while requiring limited sample sites.

### Cross-site comparison

We compiled soil thickness, hillslope curvature and total SOC stocks in Table 1 using Data Thief III^84^ for comparable datasets. These sites were all dominated by slow diffuse soil creep processes and had relatively low episodic soil movement events but varied in lithology, biology, and climate. We resampled from the original 1 m DEM to a larger 5 m resolution because most of the other datasets were collected at this scale. We compared SOC stocks to curvature and soil thickness for all sites, and evaluated the resulting functions on significance at *α* = 0.05 for all statistical tests. Residuals were examined for normality and structure as well as spatial dependence.

## Supplementary information

Supplementary Information

## References

[1] Bradford MA (2016). Managing uncertainty in soil carbon feedbacks to climate change. Nature Climate Change.

[2] Cadule P (2010). Benchmarking coupled climate‐carbon models against long‐term atmospheric carbon dioxide measurements. Global Biogeochemical Cycles.

[3] Falloon P, Jones CD, Ades M, Paul K (2011). Direct soil moisture controls of future global soil carbon changes: An important source of uncertainty. Global Biogeochemical Cycles.

[4] Friedlingstein P (2006). Climate-carbon cycle feedback analysis: Results from the C(4)MIP model intercomparison. Journal of Climate.

[5] Hopkins FM, Torn MS, Trumbore SE (2012). Warming accelerates decomposition of decades-old carbon in forest soils. Proceedings of the National Academy of Sciences.

[6] Jones C (2005). Global climate change and soil carbon stocks; predictions from two contrasting models for the turnover of organic carbon in soil. Global Change Biology.

[7] IPCC. *Climate Change 2013: The Physical Science Basis. Contribution of Working Group I to the Fifth Assessment Report of the Intergovernmental Panel on Climate Change* (eds Stocker, T. F. *et al*.) (Cambridge University Press, 2013).

[8] Kirschbaum MUF (2000). Will changes in soil organic carbon act as a positive or negative feedback on global warming?. Biogeochemistry.

[9] Lal R (2004). Soil carbon sequestration impacts on global climate change and food scarcity. Science.

[10] Todd-Brown KEO (2013). Causes of variation in soil carbon simulations from CHIP5 Earth system models and comparison with observations. Biogeosciences.

[11] Pelletier JD (2016). A gridded global data set of soil, intact regolith, and sedimentary deposit thicknesses for regional and global land surface modeling. Journal of Advances in Modeling Earth Systems.

[12] Fontaine S (2007). Stability of organic carbon in deep soil layers controlled by fresh carbon supply. Nature.

[13] Jobbágy EG, Jackson RB (2000). The vertical distribution of soil organic carbon and its relation to climate and vegetation. Ecological Applications.

[14] Rumpel C, Kögel-Knabner I (2011). Deep soil organic matter—a key but poorly understood component of terrestrial C cycle. Plant Soil.

[15] Mehler K, Schöning I, Berli M (2014). The importance of rock fragment density for the calculation of soil bulk density and soil organic carbon stocks. Soil Science Society of America Journal.

[16] Jackson RB (2017). The ecology of soil carbon: pools, vulnerabilities, and biotic and abiotic controls. Annual Review of Ecology, Evolution, and Systematics.

[17] Batjes NH (1996). Total carbon and nitrogen in the soils of the world. European Journal of Soil Science.

[18] Eswaran H, Van Den Berg E, Reich P (1993). Organic carbon in soils of the world. Soil Science Society of America Journal.

[19] Post WM, Emanuel WR, Zinke PJ, Stangenberger AG (1982). Soil carbon pools and world life zones. Nature.

[20] Kunkel ML, Flores AN, Smith TJ, McNamara J, Benner SG (2011). A simplified approach for estimating soil carbon and nitrogen stocks in semi-arid complex terrain. Geoderma.

[21] Garcia-Pauusas J (2007). Soil organic carbon storage in mountain grasslands of the Pyrenees: effects of climate and topography. Biogeochemistry.

[22] Tsui CC, Chen ZS, Hsieh CF (2004). Relationships between soil properties and slope position in a lowland rain forest of southern Taiwan. Geoderma.

[23] Fissore C (2017). Influence of topography on soil organic carbon dynamics in a Southern California grassland. Catena.

[24] Doetterl S, Six J, Van Wesemael B, Van Oost K (2012). Carbon cycling in eroding landscapes: geomorphic controls on soil organic C pool composition and C stabilization. Global Change Biology.

[25] Thompson JA, Kolka RK (2005). Soil carbon storage estimation in a forested watershed using quantitative soil-landscape modeling. Soil Science Society America Journal.

[26] Hengl T, Heuvelink GBM, Rossiter DG (2007). About regression-kriging: From equations to case studies. Computers & Geosciences.

[27] Wang L, Wu J, Liu Y, Huang H, Fang Q (2009). Spatial variability of micronutrients in rice grain and paddy soil. Pedosphere.

[28] Malone BP, McBratney AB, Minasny B, Laslett GM (2009). Mapping continuous depth functions of soil carbon storage and available water capacity. Geoderma.

[29] Meersmans J, Van Wesemael B, De Ridder F, Van Molle M (2009). Modelling the three-dimensional spatial distribution of soil organic carbon (SOC) at the regional scale (Flanders, Belgium). Geoderma.

[30] Minasny, B., McBratney, A. B., Malone, B. P. & Wheeler, I. Digital mapping of soil carbon. Advances in Agronomy 1–47 (2013).

[31] Stein A, Corsten LCA (1991). Universal kriging and cokriging as a regression procedure. Biometrics.

[32] Yang R (2016). Precise estimation of soil organic carbon stocks in the northeast Tibetan Plateau. Scientific Reports.

[33] Ivanov VY, Bras RL, Vivoni ER (2008). Vegetation-hydrology dynamics in complex terrain of semiarid areas: 2. Energy-water controls of vegetation spatiotemporal dynamics and topographic niches of favorability. Water Resources Research.

[34] Seyfried, M., Link, T., Marks, D. & Murdock, M. Soil temperature variability in complex terrain measured using fiber-optic distributed temperature sensing. *Vadose Zone Journal* **15**, vzj2015.09.0128 (2016).

[35] Patton NR, Lohse KA, Godsey SE, Crosby BT, Seyfried MS (2018). Predicting soil thickness on soil mantled hillslopes. Nature Communications.

[36] Reynolds, J. F., Maestre, F. T., Kemp, P. R., Stafford- Smith, D. M. & Lambin, E. Natural and human dimensions of land degradation in drylands: causes and consequences in *Terrestrial ecosystems in a changing world* (eds Pataki, D., Canadell, J. & Pitelka, L. F.) 247–258 (Springer, 2007).

[37] Carvalhais N (2014). Global covariation of carbon turnover times with climate in terrestrial ecosystems. Nature.

[38] Garten CT, Hanson PJ (2006). Measured forest soil C stocks and estimated turnover times along an elevation gradient. Geoderma.

[39] Pielke RA, Avissar R (1990). Influence of landscape structure on local and regional climate. Landscape Ecology.

[40] Chen LF, He ZB, Du J, Yang JJ, Zhu X (2016). Patterns and environmental controls of soil organic carbon and total nitrogen in alpine ecosystems of northwestern China. Catena.

[41] Lozano-García B, Parras-Alcántara L, Brevik EC (2016). Impact of topographic aspect and vegetation (native and reforested areas) on soil organic carbon and nitrogen budgets in Mediterranean natural areas. Science of the Total Environment.

[42] Griffiths RP, Madritch MD, Swanson AK (2009). The effects of topography on forest soil characteristics in the Oregon Cascade mountains (USA): implications for the effects of climate change on soil properties. Forest Ecology and Management.

[43] Birkeland, P. W. *Soils and Geomorphology*. Third edn, (Oxford University Press, 1999).

[44] Hurst MD, Mudd SM, Walcott R, Yoo K (2012). Using hilltop curvature to derive the spatial distribution of erosion rates. Journal of Geophysical Research: Earth Surface.

[45] Moore ID, Grayson RB, Landson AR (1991). Digital terrain modeling: a review of hydrological, geomorphological, and biological applications. Hydrological Processes.

[46] Pennock DJ, Jong ED (1987). The influence of slope curvature on soil erosion and deposition in hummock terrain. Soil Science.

[47] Stefano CD, Ferro V, Porto P, Tusa G (2000). Slope curvature influence on soil erosion and deposition processes. Water Resources Research.

[48] Yoo K, Amundson R, Heimsath AM, Dietrich WE (2005). Process-based model linking pocket gopher (Thomomys bottae) activity to sediment transport and soil thickness. Geology.

[49] Yoo K, Ronald Amundson A, Heimsath A, Dietrich WE (2006). Spatial patterns of soil organic carbon on hillslopes: Integrating geomorphic processes and the biological C cycle. Geoderma.

[50] Wang X (2018). Soil organic carbon and mineral interactions on climatically different hillslopes. Geoderma.

[51] Rosenbloom NA, Doney SC, Schimel DS (2001). Geomorphic evolution of soil texture and organic matter in eroding landscapes. Global Biogeochemical Cycles.

[52] Anderson SP (2012). How deep and how steady is Earth’s surface?. Geology.

[53] Wackett AA, Yoo K, Amundson R, Heimsath AM, Jelinski NA (2018). Climate controls on coupled processes of chemical weathering, bioturbation, and sediment transport across hillslopes. Earth Surface Processes and Landforms.

[54] Lybrand RA, Rasmussen C (2015). Quantifying climate and landscape position controls on soil development in semiarid ecosystems. Soil Science Society of America Journal.

[55] Sanderman J, Lohse KA, Baldock JA, Amundson R (2009). Linking soils and streams: Sources and chemistry of dissolved organic matter in a small coastal watershed. Water Resources Research.

[56] Godsey SE (2018). Eleven years of mountain weather, snow, soil moisture and streamflow data from the rain–snow transition zone – the Johnston Draw catchment, Reynolds Creek Experimental Watershed and Critical Zone Observatory, USA. Earth System Science. Data.

[57] Bryden, S. A. *Coupled soil moisture, soil temperature, and snow dynamics in complex terrain of a semi-arid mountainous watershed* Masters of Science thesis (University of Idaho, 2013).

[58] Nielson, T. *Application of hydrogeophysical imaging in the Reynolds Creek Critical Zone Observatory* Masters of Science thesis (Boise State University, 2017).

[59] Guo LB, Gifford RM (2002). Soil carbon stocks and land use change: a meta analysis. Global Change Biology.

[60] Lohse KA, Sanderman J, Amundson R (2013). Identifying sources and processes influencing nitrogen export to a small stream using dual isotopes of nitrate. Water Resources Research.

[61] Lohse KA, Brooks PD, McIntosh JC, Meixner T, Huxman TE (2009). Interactions between biogeochemistry and hydrologic systems. Annual Review of Environment and Resources.

[62] Webster R, Oliver MA (1992). Sample adequately to estimate variograms of soil properties. European Journal of Soil Science.

[63] Rosenbloom N, Harden JW, Neff JC, Schimel DS (2006). Geomorphic control of landscape carbon accumulation. Journal of Geophysical Research.

[64] Will, R. M. *et al*. Reynolds Creek – a collection of near-surface soil organic carbon (SOC) maps, GIS/Map Data, 10.18122/B2Q598 (2017).

[65] Will, R. M. *Mapping soil organic carbon (SOC) in a semi-arid mountainous watershed using variables from hyperspectral, lidar and traditional datasets* Masters of Science thesis (Boise State University, 2017).

[66] Noy-Meir I (1973). Desert ecosystems: Environment and producers. Annual Review of Ecology and Systematics.

[67] Boettinger, J. L., Howell, D. W., Moore, A. C., Hartemink, A. E. & Kienast-Brown, S. *Digital Soil Mapping: Bridging Research, Environmental Application, and Operation* 439 (Springer New York, 2010).

[68] Fellows A, Flerchinger G, Seyfried M, Lohse KA, Patton NR (2019). Controls on gross production in an aspen-sagebrush vegetation mosaic. Ecohydrology.

[69] Fellows A, Flerchinger G, Seyfried MS, Lohse KA (2018). Rapid recovery of mesic mountain big sagebrush gross production and respiration following prescribed fire. Ecosystems.

[70] Heimsath AM, Furbish DJ, Dietrich WE (2005). The illusion of diffusion: field evidence for depth-dependent sediment transport. Geological Society of America.

[71] Van Oost K (2012). Legacy of human-induced C erosion and burial on soil–atmosphere C exchange. Proceedings of the National Academy of Science of the United States of America.

[72] Stallard RF (1998). Terrestrial sedimentation and the carbon cycle: Coupling weathering and erosion to carbon burial. Global Biogeochemical Cycles.

[73] Harden JW (1999). Dynamic replacement and loss of soil carbon on eroding cropland. Global Biogeochemical Cycles.

[74] Liu S, Bliss N, Sundquist E, Huntington TG (2003). Modeling carbon dynamics in vegetation and soil under the impact of soil erosion and deposition. Global Biogeochemical Cycles.

[75] Kirkels FMSA, Cammeraat ELH, Kuhn NJ (2016). The fate of soil organic carbon upon erosion, transport and deposition in agricultural landscapes: A review of different concepts. Geomorphology.

[76] Doetterl S (2016). Erosion, deposition and soil carbon: a review of process-level controls, experimental tools and models to address C cycling in dynamic landscapes. Earth-Science Reviews.

[77] Pansze, A. J. Geology and ore deposits of the Silver City-DeLamar-Flint region, Owyhee County, Idaho. **79** (Idaho Bureau of Mines and Geology, 1975).

[78] McIntyre, D. H. Cenozoic geology of the Reynolds Creek Experimental Watershed, Owyhee County, Idaho. **P-151** (Idaho Bureau of Mines and Geology, 1972).

[79] Vincent KR, Chadwick OA (1994). Synthesizing bulk density for soils with abundant rock fragments. Science Society of America Journal.

[80] Patton NP, Lohse KA, Seyfried MS, Will R, Benner SG (2019). Lithology and coarse fraction adjusted bulk density estimates for determining total organic carbon stocks in dryland soils. Geoderma.

[81] Boone, R. D., Grigal, D. F., Sollins, P. & Armstrong, D. E. Soil sampling, preparation, archiving, and quality control in *Standard Soil Methods for Long-term Ecological Research* (ed. Robertson, P) (1999).

[82] Blake, G. R. & Hartge, K. H. Bulk density in *Methods of Soil Analysis: Part I—Physical and Mineralogical Methods* (ed. Klute, A) 363–375 (SSSA, 1986).

[83] Zevenbergen LW, Thorne CR (1987). Quantitative analysis of land surface topography. Earth Surface Processes and Landforms.

[84] Tummers, B. DataThief III, http://datathief.org/, (2006).

