# Supplementary material for: Topographic controls of soil organic carbon on soil-mantled landscapes
Source: Sci Rep. 2019 Apr 23;9:6390. doi: 10.1038/s41598-019-42556-5 (PMC6478868; doi:10.1038/s41598-019-42556-5)
Supplement: Supplementary file 1 — Supplementary Information [file 41598_2019_42556_MOESM1_ESM.pdf]

Supplementary Information

for

Topographic controls on soil organic carbon on soil mantled landscapes

Authors: Nicholas R. Patton[1+†], Kathleen A. Lohse[1,2+], Mark S. Seyfried[3], Sarah E. Godsey[1], Susan B. Parsons[2]

Affiliations:

[1]Department of Geosciences, Idaho State University, Pocatello, ID 83209,

[2]Department of Biological Sciences, Idaho State University, Pocatello, ID 83209,

[3]Agricultural Research Service, Northwest Watershed Research Center, 800 Park Blvd., Plaza IV, Suite 105 Boise, Idaho 83712, USA.

*Correspondence to: klohse@isu.edu

[+] these authors contributed equally to this work

[†] Current address: School of Earth and Environmental Sciences, University of Queensland, St Lucia, 4072, Qld, Australia

This document file includes:

Supplementary Figure 1

Supplementary Table 1

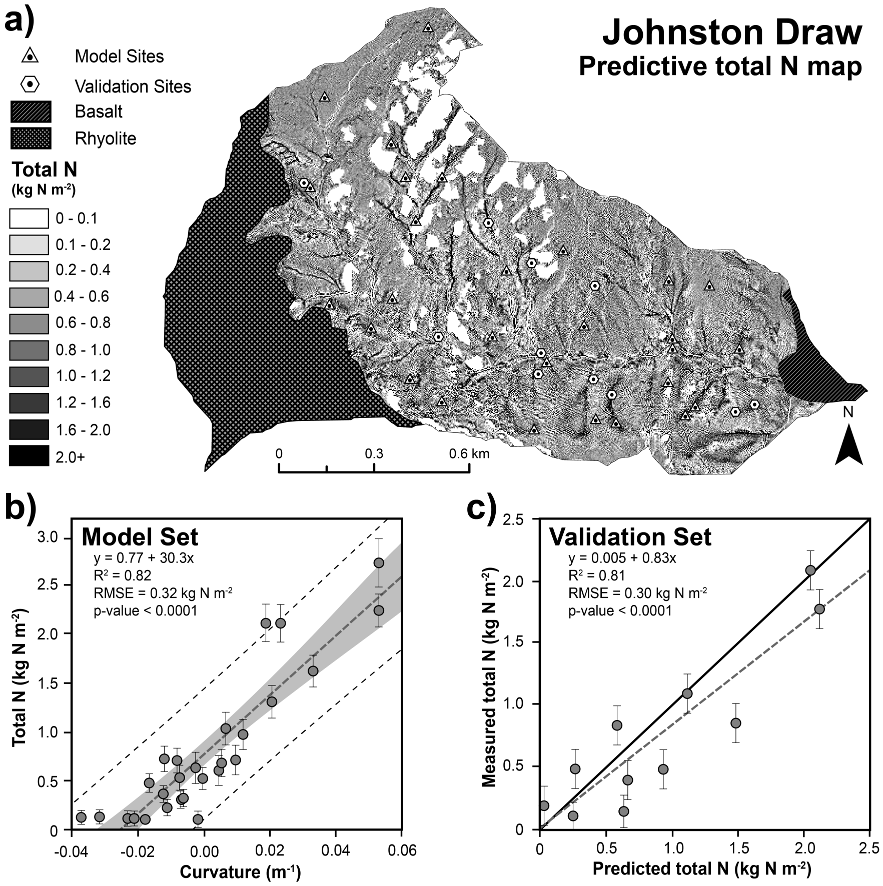

Supplementary Figure 1: Soil total nitrogen (N)-curvature relationship. (a) A 3 m predictive total nitrogen map of Johnston Draw. Figure depicts the location of the model (triangles) and validation sites (hexagons) within the soil mantled, granitic portion of Johnston Draw watershed. (b) Curvature to total nitrogen (curvature-N) function for the entire watershed showing a positive linear relationship (dashed line) bounded by 95% confidence limits (dotted line) (n=28, R^2 = 0.82, RMSE = 0.32 kg N m^{-2}, $p < 0.0001$). Curvature-N functions were derived from a model set consisting of 70% of all sites. (c) The remaining 30% of sites were utilized as a validation set and evaluated against a 1:1 line (solid line) in predicted versus measured graph (dashed line). Total N validation sets showed that predicted total-profile N agreed well with observed values (n=11, R^2 = 0.81, RMSE = 0.30 kg N m^{-2}, $p < 0.0001$).

Supplementary Table 1: Field soil descriptions of representative soil profiles on convergent and divergent topography and south and north facing aspects at different locations (Eastings and Northings, respectively, using NAD 83 UTM Zone 11T). Boundaries (BDRY) determined by ocular inspection, Munsell color (hue (H), value (V), chroma (C) was determined when soil was dry (D) and moist (M), structure refers to the grade (0– 3, structureless to strong), size (vf, very fine; f, fine; m, medium; co, coarse) and type (gr, granular; abk, angular blocky, sbk, subangular blocky; m, massive, pl, platy), and roots and pores refers to the grade (1-3 few to many) and size (vf, very fine; f, fine; m, medium; co, coarse), bioturbance, absence/presence, and vegetation.

Site	*Location and Topographic position*	*Soil Horizon*	*Depth (cm)*	*B_{DRY}*	*Color (dry)*	*Color (wet)*	*Structure*	*Roots*	*Pores*	*Clay Films*	*Bioturbation*	*Vegetation*
Johnston Draw (JDT 2g NRP)	517,404.683 4,774,666.411 (m)	A1	0-15	CW	7.5YR 4/4	7.5YR 3/3	1Gr Vf-F	3 Vf-F	No	No	No	*Artemisia tridentate* ssp. *wyomingensis*
		A2	15-30	GW	7.5YR 4/4	7.5YR 3/3	2Sbk Vf-M	3 Vf	No	No	No	
		CR	30-90	D Ir	7.5YR 6/6	7.5YR 6/5	Massive	No	No	No	No	*Juniperus occidentalis*
	South-facing Divergent Topography	R	90+	-	-	-	-	-	No	No	No	*Purshia stansburyana*
Johnston Draw (JDT 3e NRP)	517,505.977 4,774,359.875 (m)	A1	0-30	CS	10YR 3/2	10YR 2/1	3Sbk Vf-M	3 Vf	1 Vf	No	No	*Artemisia tridentate* ssp. *wyomingensis*
		A2	30-92	CS	10YR 3/3	10YR 2/2	3Sbk M-Co	3 Vf	1 Vf	No	No	*Symphoricarpos parishii*
	North-facing Convergent Topography	AC1	92-148	CS	10YR 4/4	10YR 3/3	3Sbk M-Co	3 Vf	1 Vf	No	Yes	
		AC2	148-175	CS	10YR 4/4	10YR 3/4	3Sbk Co-VC	3 Vf	1 Vf	No	Yes	
		CA1	175-200	CS	10YR 4/4	10YR 3/4	3Sbk Vf-M	3 Vf	1 Vf	No	Yes	
		CA2	200-213	CS	10YR 5/4	10YR 3/6	3Sbk Vf-M	3 Vf	1 Vf	No	No	
		CR	+213	-	-	-	-	-	-	No	No	
Johnston Draw (JDT 5c NRP)	516,800.011 4,774,750.710 (m)	A	0-18	C Ir	10YR 5/3	10YR 3/3	1Gr Vf-F	3 Vf	No	No	No	*Artemisia tridentate* ssp. *wyomingensis*
	South-facing Divergent Topography	R	+18	-	-	-	-	-	-	No	No	*Cercocarpus ledifolius*
Johnston Draw (JDT 5d NRP)	517,103.179 4,774,994.265 (m)	A	0-10	CS	10YR 4/3	10YR 3/2	1Gr Vf	3 Vf	3 Vf	No	No	*Artemisia tridentate* ssp. *wyomingensis*
		AC	10-25	GS	10YR 4/3	10YR 2/2	2Sbk Vf-F	3 Vf	3 Vf	No	No	*Cercocarpus ledifolius*
	South-facing Planar Topography	C	25-75	GS	10YR 4/3	10YR 3/3	2Sbk Vf-F	1 Vf	1 Vf	No	No	
		R	+75	-	-	-	-	-	-	No	No	
Johnston Draw (JDT 5e NRP)	516,731.729 4,774,660.012 (m)	A1	0-12	GS	10YR 2/1	10YR 2/1	1Gr F	3 Vf-F	No	No	Yes	*Populus tremuloides*
		A2	12-52	GW	10YR 3/1	10YR 2/1	3Sbk M-VC	3 Vf-VC	1 Vf	No	Yes	
	North-facing Planar Topography	A3	52-78	CS	10YR 4/3	10YR 3/3	2Sbk M-VC	2 Vf-VC	2 Vf	No	Yes	
		Bw	78-94	G Ir	10YR 4/3	10YR 3/3	2Sbk Co-VC	1 F	3 Vf	No	Yes	
		C	94-132	CS	10YR 6/4	10YR 4/6	2Sbk M-VC	3 Vf-F	1 Vf	No	Yes	
		CR	+132	-	-	-		-	-	No	No	
Johnston Draw (JDT 6k NRP)	516,601.553 4,774,732.500 (m)	A	0-17	C Ir	10YR 4/2	10YR 2/1	1Gr Vf-F		3 Vf	No	No	*Artemisia arbuscula* *Artemisia tridentate* ssp. *wyomingensis*
	North-facing Divergent Topography	R	+17	-	-	-	-		-	No	No	

Supplementary Table 1 continuation: Laboratory soil descriptions of representative soil profiles on convergent and divergent topography and south and north facing aspects. Particle size distribution as sand, silt, clay, bulk density (BD), % coarse fraction (CF) were determined with modified hydrometer where the sand fraction was sieved through a 0.53 um sieve. BD was determined using a CF adjusted bulk density method described by Patton *et al.*[80]. Soil organic matter (%) is based on loss on ignition (dried samples combusted at 8 hr at 450 °C), soil total N % and soil organic carbon % based on methods detailed in paper. Soil pH and electrical conductivity (EC) were determined in the field on 2:1 water to soil slurries after sitting for 60 minutes. Presence/absence of carbonates (EFF) determined by effervescence (application of 10% HCl to soil).

Site	*Soil Horizon*	*Depth (cm)*	*Sand %*	*Silt %*	*Clay %*	*BD (g/cm^3)*	*% CF (wt/wt)*	*Soil Organic Material (%)*	*% Soil Total Nitrogen (wt/wt)*	*% Soil Organic Carbon (wt/wt)*	*pH (2:1)*	*EC (µS/cm)*	*EFF*
Johnston Draw (JDT 2g NRP)	A1	0-15	82	8	10	1.21	19	2.1	0.08	0.92	5.98	44.30	No
	A2	15-30	83	6	11	1.12	27	1.2	0.05	0.43	6.13	2.50	No
	CR	30-90	88	6	6	-	29	0.4	0.01	0.09	6.65	11.23	No
	R	90+	-	-	-	-	-	-	-	-	-	-	No
Johnston Draw (JDT 3e NRP)	A1	0-30	66	11	23	1.37	18	9.8	0.33	5.74	5.82	89.23	No
	A2	30-92	53	24	24	1.32	18	7.6	0.26	3.65	5.49	23.71	No
	AC1	92-148	61	18	21	1.48	27	3.5	0.13	1.58	5.47	12.23	No
	AC2	148-175	67	15	19	1.53	30	3.1	0.07	0.98	5.55	11.68	No
	CA1	175-200	67	13	20	1.67	36	2.9	0.04	0.59	5.86	11.55	No
	CA2	200-213	74	11	15	1.39	35	1.8	0.01	0.24	5.65	8.37	No
	CR	+213	-	-	-	-	-	-	-	-	-	-	No
Johnston Draw (JDT 5c NRP)	A	0-18	66	15	19	1.44	39	2.9	0.09	1.03	6.5	44.07	No
	R	+18	-	-	-	-	-	-	-	-	-	-	No
Johnston Draw (JDT 5d NRP)	A	0-10	74	13	13	0.93	20	5.1	0.17	2.28	5.19	142.77	No
	AC	10-25	78	13	9	1.09	24	4.4	0.12	1.48	5.92	43.80	No
	C	25-75	78	10	12	1.47	32	2.5	0.06	0.48	6.01	42.27	No
	R	+75	-	-	-	-	-	-	0.02	0.19	-	-	No
Johnston Draw (JDT 5e NRP)	A1	0-12	53	33	13	1.31	19	12.1	0.54	7.23	5.62	713.67	No
	A2	12-52	45	40	14	1.12	17	10.1	0.33	4.16	5.56	102.47	No
	A3	52-78	42	41	17	1.21	27	7.4	0.11	1.23	6.01	16.87	No
	Bw	78-94	46	34	19	1.27	36	4.4	0.05	0.44	5.57	13.23	No
	C	94-132	69	16	14	1.39	55	2.3	0.04	0.33	5.50	22.07	No
	CR	+132	-	-	-		-	-	0.00	0.05	-	-	No
Johnston Draw (JDT 6k NRP)	A	0-17	54	28	18	1.03	50	7.7	0.16	2.16	6.62	191.90	No
	R	+17	-	-	-	-	-	-	-	-	-	-	No
